# Decontamination Efficacy and Skin Toxicity of Two Decontaminants against *Bacillus anthracis*

**DOI:** 10.1371/journal.pone.0138491

**Published:** 2015-09-22

**Authors:** Chad W. Stratilo, Melissa K. F. Crichton, Thomas W. Sawyer

**Affiliations:** 1 Biological Threat Defence Section, Defence Research and Development Canada – Suffield Research Centre, Medicine Hat, Alberta, Canada; 2 Casualty Management Section, Defence Research and Development Canada – Suffield Research Centre, Medicine Hat, Alberta, Canada; University of Connecticut, UNITED STATES

## Abstract

Decontamination of bacterial endospores such as *Bacillus anthracis* has traditionally required the use of harsh or caustic chemicals. The aim of this study was to evaluate the efficacy of a chlorine dioxide decontaminant in killing *Bacillus anthracis* spores in solution and on a human skin simulant (porcine cadaver skin), compared to that of commonly used sodium hypochlorite or soapy water decontamination procedures. In addition, the relative toxicities of these decontaminants were compared in human skin keratinocyte primary cultures. The chlorine dioxide decontaminant was similarly effective to sodium hypochlorite in reducing spore numbers of *Bacillus anthracis* Ames in liquid suspension after a 10 minute exposure. After five minutes, the chlorine dioxide product was significantly more efficacious. Decontamination of isolated swine skin contaminated with *Bacillus anthracis* Sterne with the chlorine dioxide product resulted in no viable spores sampled. The toxicity of the chlorine dioxide decontaminant was up to two orders of magnitude less than that of sodium hypochlorite in human skin keratinocyte cultures. In summary, the chlorine dioxide based decontaminant efficiently killed *Bacillus anthracis* spores in liquid suspension, as well as on isolated swine skin, and was less toxic than sodium hypochlorite in cultures of human skin keratinocytes.

## Introduction

*Bacillus* endospores are resistant to destruction by measures that are effective in decontaminating most biological agents [1]. Various decontamination approaches have been evaluated for efficacy against *Bacillus* spores, including *B*. *anthracis*, on various surfaces or in suspension [2, 3]. The measures commonly used to kill *Bacillus* endospores are often associated with harsh environmental conditions such as ultra violet light, wet and dry heat, radiation, or exposure to chemicals [2–5]. Gaseous forms of methyl bromide, ethylene oxide, chlorine dioxide, formaldehyde and hydrogen peroxide, have been shown to inactivate *Bacillus* spores [6–8].

Sodium hypochlorite (5250–6000 ppm free chlorine), has been shown to be effective in killing *Bacillus* spores [9]. In liquid suspension testing, spore numbers are greatly reduced (>5 logs) within 2–3 minutes of contact with 0.5% sodium hypochlorite[8]. However, a few viable spores do remain even after 10–30 minutes of contact time depending on the initial spore concentration [8]. Correcting the sodium hypochlorite solution to a pH of less than 7, has shown to increase effectiveness in some experiments [10–12]. Sodium hypochlorite, is highly effective against *Bacillus* spores but is not suited for personal decontamination due to toxic effects [13]. Commercial sodium hypochlorite is corrosive and may irritate the skin causing inflammation and blisters [9]. Sodium hypochlorite has been shown to be cytotoxic and adversely affects wound healing in animal models [14, 15]. Tissue toxicity, both in vitro and in vivo, has been observed at concentrations of 0.25% sodium hypochlorite [16].

Liquid chlorine dioxide has been shown to be sporicidal [17]. Chlorine dioxide products rapidly destroy bacterial spores, and vegetative bacteria [18]. A five‐log reduction in bacterial populations for several strains of bacteria is achievable with a 60 second contact time, at relatively low concentrations compared to other biocides [19]. Chlorine dioxide has been suggested to be a size selective antimicrobial agent, killing micron sized organisms rapidly but causing much less harm to larger organisms compared to hypochlorite solutions [20]. Disinfection of living tissues with aqueous chlorine dioxide solutions appears to be quite safe as bacteria can be killed using low concentrations of chlorine dioxide and short contact times, while much higher concentrations and contact times are required to cause damage to tissue [20].

The events of 2001, where *B*. *anthracis* laced letters caused infection of at least 22 people (five deaths) and contaminated at least 42 buildings demonstrated the ease of deliberate dissemination of this organism, as well as its mass casualty potential [21]. Current guidelines recommend decontamination of exposed individuals with water or soap and water [22, 23]. This measure would reduce the number of spores on the exposed persons, but would have no effect on spore viability and also contaminate the effluent with viable spores. Many current products, including alcohol wipes and rubs fail to reduce or effectively remove *Bacillus* spores [24]. A 0.5% sodium hypochlorite solution has been suggested as a decontaminant for chemical warfare and biological agents [13, 25].

The aim of this study was to evaluate the efficacy of a chlorine dioxide decontaminant in killing *B*. *anthracis* spores in solution and on a human skin simulant (porcine cadaver skin), compared to that of commonly used sodium hypochlorite or soapy water decontamination procedures. In addition, the relative toxicities of these decontaminants in cell cultures of human origin were compared.

## Materials and Methods

*B*. *anthracis* strains were obtained from the Animal Disease Research Institute (Lethbridge, Alberta, Canada) and the United States Army Medical Research Institute for Infectious Diseases (Frederick, Maryland, USA). All procedures involving the fully virulent strain; *B*. *anthracis* (Ames) were carried out in certified biosafety level 3 facilities accredited by the Canadian Food Inspection Agency and Public Health Agency of Canada. Sodium hypochlorite solutions were prepared from commercially available bleach, dilutions were made with water and the chlorine concentration was established using Activate high level chlorine test strips (Cole-Parmer). The chlorine dioxide decontaminant was a gift from Vital Solutions, LLC, West Palm Beach, Florida. This test decontaminant is similar to a commercially available product called Vital Oxide® (Vital Solutions, LLC, West Palm Beach, Florida) which is a disinfectant composed of a stabilized form of chlorine dioxide, betaine hydrochloride and propylene glycol[26]. This product tested is not currently commercially available, it should be noted the commercially available Vital Oxide does not have the same level of sporicidal activity as the product disinfectant tested. RODAC Trypticase^TM^ Soy Agar with Lecithin and Polysorbate 80 (Becton, Dickinson and Company) were used for surface sampling. D/E (Dey/Engley) Neutralizing Broth (Fisher) was used to quench the disinfectants tested.

Swine skin was obtained from castrated male York-Landrace cross swine (~20 kg) that had been euthanized after short-term experimental studies (TWS-11-1-1). The conduct of this latter research (not related to this study) adhered to the “Guide to the Care and Use of Experimental Animals” and “The Ethics of Animal Experimentation” published by the Canadian Council on Animal Care.

### Bacterial Cultures and Spore Preparation

The veterinary vaccine strain of *B*. *anthracis* Sterne and the fully virulent strain of *B*. *anthracis* Ames were grown in 2 ml cultures of Luria Broth (LB) overnight. 0.5 ml of each culture was used to inoculate larger volumes of Casein–Casein–Yeast (CCY) liquid medium which was incubated for 96 hours at 37°C on an orbital shaker (200 rpms) [27]. The cells were checked for sporulation by microscopic examination, then harvested by centrifugation at 3500 x *g* for 30 min and rinsed with cold, sterile, ultrapure reverse osmosis (RO) water. The spores pelleted by centrifugation were resuspended in 50% ethanol and incubated at room temperature for 2 hours. The spores were again harvested by centrifugation at 5,000 x *g* for 30 min and washed 3 times with cold, sterile, ultrapure RO water. This stock spore suspension was stored at 4°C. The spores were quantified by serially diluting the stock and plating 100 μl aliquots on LB plates, in triplicate. The numbers of spores based on colony forming units (CFU ml^-1^) were counted following an overnight incubation at 37°C.

### Exposure of spores to disinfectant over time via suspension testing

*B*. *anthracis* spore stock solutions (10^7^–10^8^CFU for Sterne and 10^6^–10^7^CFU for Ames) were vortexed and added to a tube containing an aliquot of the test chemical decontaminant at a specified concentration and vortexed to mix. After the specified spore exposure time elapsed, the test tube was vortexed for 10 to 20 s, and a sample was placed in a test tube containing PBS or DE broth. D/E neutralizing broth was used to quench the activity of the disinfectant at given time points. The samples were serially diluted as required, to be within a countable range of 15–300 CFU, and plated in triplicate using a petri dish spinner. The petri dish was covered, inverted and incubated >24 h at 37°C. The procedure was repeated in at least triplicate for all predetermined spore exposure times of 2, 5, 10 and 30 minutes for the chlorine dioxide decontaminant, a 10% dilution of household bleach equivalent to a 0.5% sodium hypochlorite solution and PBS (control).

When initial suspension testing using *B*. *anthracis* Sterne yielded no viable colonies on the count plates an alternate enumeration method was used that analyzed the entire volume of suspension. The ten milliliter suspension volume was vacuum filtered through a .22 μm filter and washed with PBS. The filter was placed on a LB plate and incubated overnight at 37°C. The plates were examined for growth +/-.

### Decontamination of human skin simulant with chlorine dioxide decontaminant

Sections (~8 x 8 cm) of full thickness porcine skin that had been shaved were used for testing the efficacy of the test decontaminants (chlorine dioxide and soapy water) on skin. The porcine skin was fixed to plastic trays with tacks on each corner. *B*. *anthracis* spores (~8 x 10^7^ CFU in a 100 μl volume) were spread on the skin and allowed to dry for 30 minutes. Spore loads on the skin were established using contact RODAC Trypticase^TM^ Soy Agar with Lecithin and Polysorbate 80 (Becton, Dickinson and Company) [28]. Contaminated skin was treated with chlorine dioxide decontamination solution or soapy water for a total contact time of 5 minutes. The swine skin was initially scrubbed, with consistent gentle pressure between samples, with a soft bristled tooth brush for 30 seconds to lift the spores from the skin. Spore loads on treated skin were established using contact RODAC plates. Cleaning solutions and brushes were sampled to establish spore load on materials used after cleaning. RODAC plates were examined for the presence of growth after 16 hours. Spore loads after treatments were compared to spore loads before treatment.

### Keratinocyte Cell Culture Treatment

Early passage human neonatal skin keratinocyte cultures (Life Technologies, Grand Island, NY) were used for cytotoxicity testing at ~40–60% confluence (3–4 days after seeding) as previously described [29]. At the time of treatment, the culture medium volume was adjusted to 50 ul and an equal volume of test decontaminant in fresh Keratinocyte-Serum-free medium (KSFM) was added. The decontaminants were serially diluted in KSFM immediately before use, and then incubated with the cells for 1, 4 or 24 hours before being aspirated, and replaced with fresh medium containing no test product. Cytotoxicity was assessed using alamarBlue (AccuMed International Inc., Westlake, OH) at 24 hr. The dye was added for the last 3 hr of the test period and the fluorescence (570 nm–600 nm) was read on a Thermomax titerplate reader (Molecular Devices, Sunnyvale, CA). After the 24 hr cytotoxicity determination, the non-toxic dye was removed and the cultures were refreshed with fresh medium and tested again at 48 hr. Median lethal concentration (LC_50_) values were determined graphically from experiments utilizing 6 wells per data point. Experiments were replicated four times.

## Results

**Fig 1:**
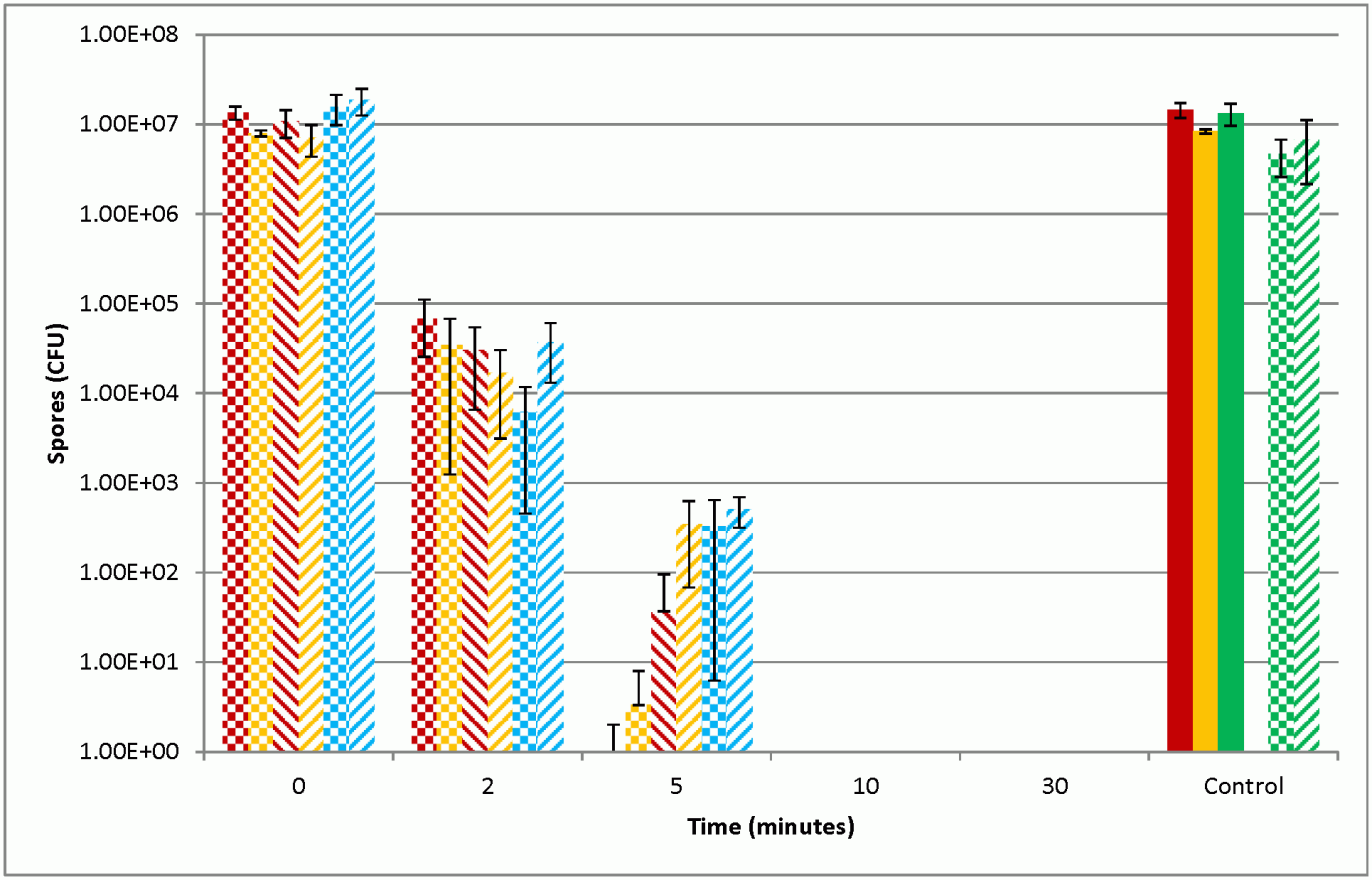
Recovery of *B*. *anthracis* spores after exposure to decontaminants. Recovery of *B*. *anthracis* Sterne spores (Red) or Ames spores (Yellow) after exposure to test decontaminants over specified periods of time. Sterne spores were quenched using DE broth (blue) after exposure to the decontaminants. Untreated controls (solid bars), chlorine dioxide based test decontaminant (checker bars), 0.5% sodium hypochlorite (striped bars). Quench control samples (Green) were DE broths incubated with each decontaminant prior to Sterne spore addition or in DE broth alone (solid bar). Data are presented as means (n = 3) CFU ml^-1^ of spores recovered after specified contact times with decontaminants.

Suspension testing was used to determine the efficacy of the sporicides. After five minutes of contact time with the chlorine dioxide decontaminant spore reduction was >log7 for Sterne or >log5 for Ames. The chlorine dioxide decontaminant reduced spore loads to <log 1 after five minutes of exposure regardless of the strain of *B*. *anthracis* used compared to a PBS control. The time to achieve kill levels below the limit of detection was superior with the chlorine dioxide decontaminant when compared to 0.5% sodium hypochlorite that was not pH corrected. After 5 minutes of contact time with the chlorine dioxide product no spores were recovered at the limit of detection. This result was also achieved after 10 minutes of contact time with 0.5% sodium hypochlorite (Fig 1). These results were comparable between the veterinary vaccine strain of *B*. *anthracis* Sterne and the fully virulent strain of *B*. *anthracis* Ames (Fig 1) used in the study.

The results from the analysis of the entire volume of the ten milliliter suspension that was filtered through a .22 μm filter and washed with PBS support the dilution results. Contact time greater than five minutes resulted in no colonies being observed on the filters from the chlorine dioxide decontaminant (data not shown). After ten minutes of contact time no colonies were observed on filters from the bleach or the chlorine dioxide decontaminant (Data not shown).

**Fig 2:**
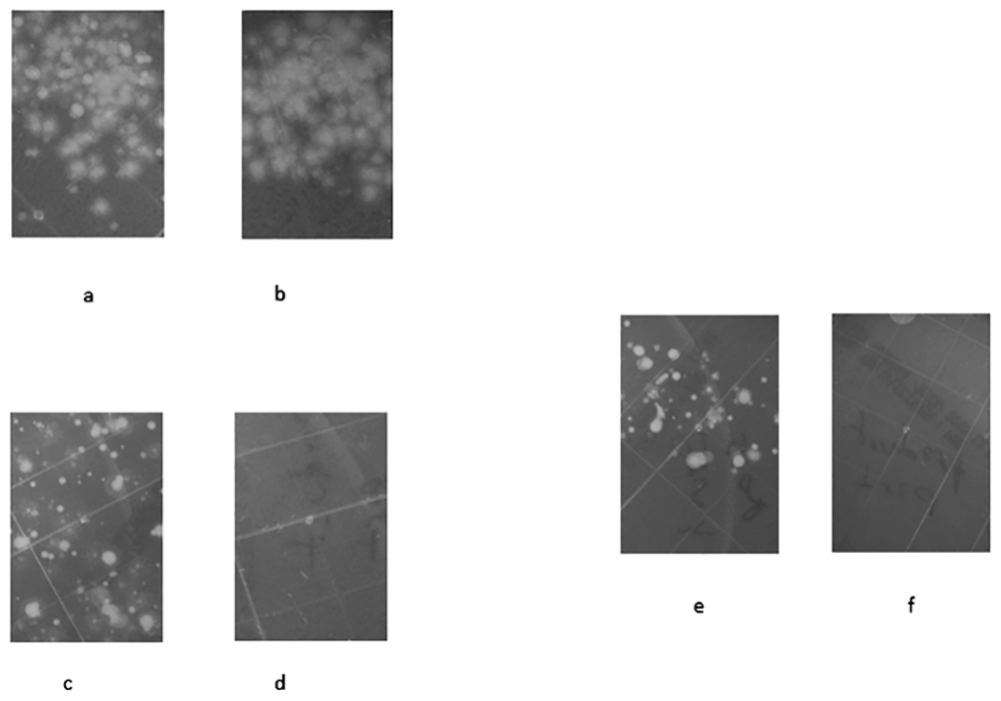
RODAC plate samplings of swine skin contaminated with *B*. *anthracis* spores. Photos of RODAC plate samplings of swine skin contaminated with *B*. *anthracis* spores. a) Pre-soapy water exposure. b) Pre-chlorine dioxide decontaminant exposure. c) Post- 30 second scrub and 5 minute soapy water total contact time. d) Post- 30 second scrub and 5 minute total contact time. e) Soapy water scrub brush after scrubbing skin for 30 seconds with soapy water. f) Chlorine dioxide decontaminant scrub brush after scrubbing skin for 30 seconds with chlorine dioxide decontaminant.

The chlorine dioxide decontaminant was effective in decontaminating swine skin after exposure to 10^7^ *B*. *anthracis* spores (Sterne strain). Sampling using RODAC plates showed heavy contamination of the swine skin prior to decontamination (Fig 2a and 2b). Sampling of the skin after scrubbing in soapy water showed substantial numbers of spores remaining on the skin (Fig 2c). In contrast, decontamination of swine skin with the chlorine dioxide decontaminant (with scrubbing), resulted in no spores being detected on the skin (Fig 2d). The effluent and the brushes used were also sampled for the presence of spores using RODAC contact plates. The soapy water solution had a large number of spores present, where only dilution impacted the number of spores ml^-1^ sampled compared to the original contaminated skin. The brush used for scrubbing the skin also showed heavy contamination when sampled using contact plates (Fig 2e). The effluent containing the chlorine dioxide decontaminant showed no spore growth plated either diluted or undiluted. The scrub brush used with the chlorine dioxide decontaminant also provided no viable spores when sampled (Fig 2f).

**Fig 3:**
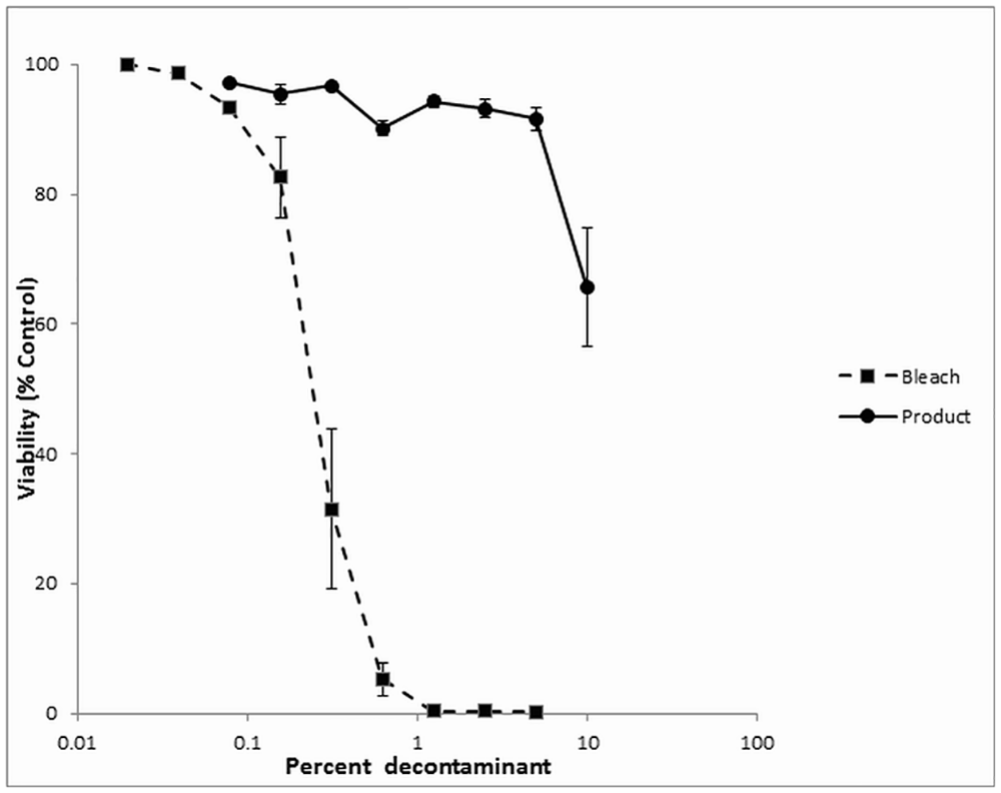
Toxicity of sodium hypochlorite and chlorine dioxide decontaminant in human skin keratinocytes cells. Toxicity of sodium hypochlorite (0.5%) and chlorine dioxide decontaminant in human skin keratinocytes cells. Just-confluent keratinocyte cultures were treated with varying concentrations of sodium hypochlorite (squares) or chlorine dioxide based decontaminant (circles), diluted in culture medium and incubated for 1 hr before being removed and replaced with fresh culture medium containing no decontaminant. Cytotoxicity was determined at 24 h using the alamarBlue^TM^ cytotoxicity assay. Results represent the mean +/- SEM of four experiments.

The relative toxicities of sodium hypochlorite and the chlorine dioxide decontaminant were assessed in human skin keratinocyte cultures. Fig 3 shows the toxicity of the two products when the cells were exposed to the decontaminant for 1 hr before being removed and replaced with fresh medium. The chlorine dioxide decontaminant (LC50 >10%, v/v) was almost two orders of magnitudes less toxic than the sodium hypochlorite (LC50 = 0.14%, v/v).

**Fig 4:**
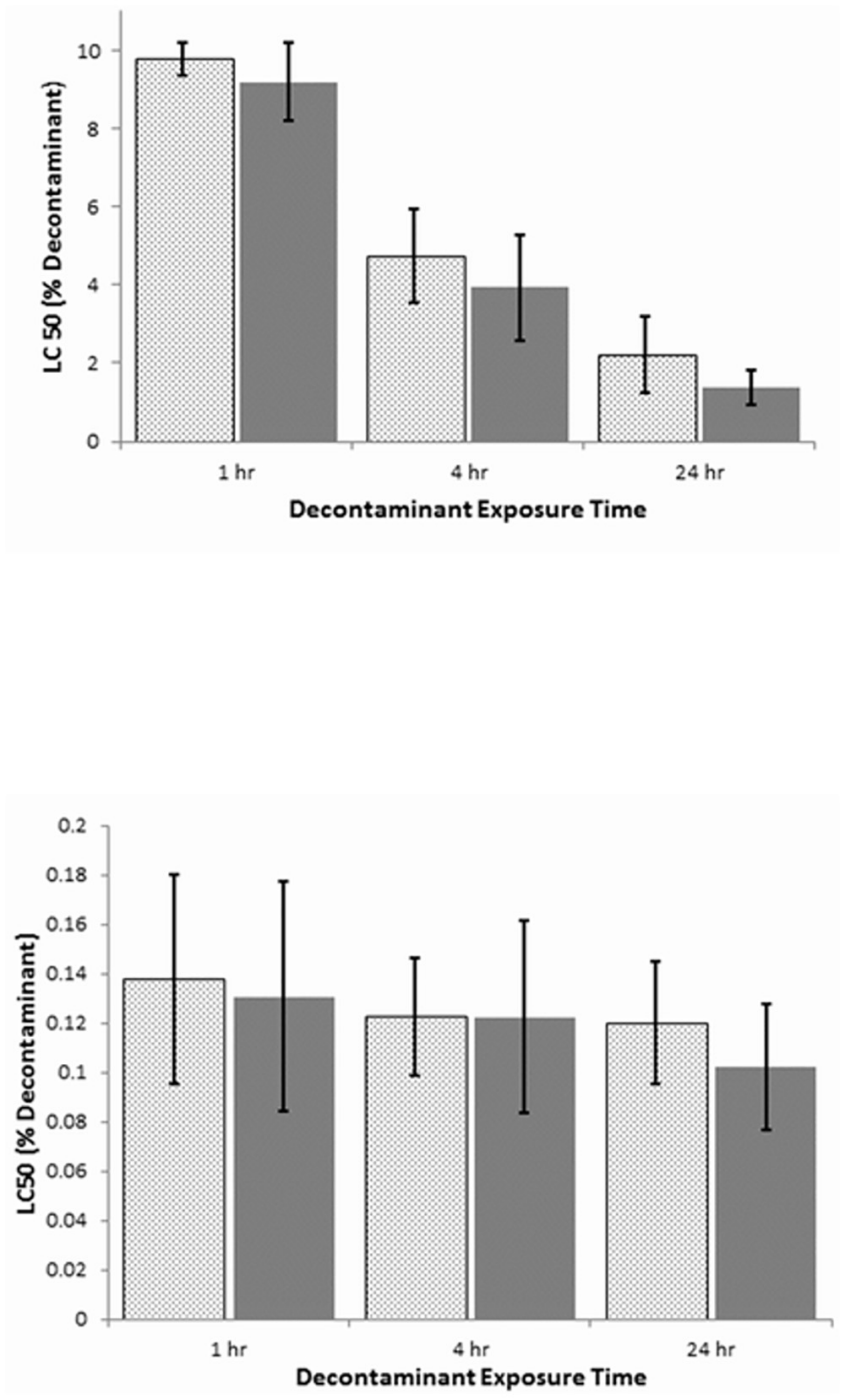
Effect of exposure time on the toxicity of chlorine dioxide decontaminant (a) and sodium hypochlorite (b) in human skin keratinocyte cells. Effect of exposure time on the toxicity of chlorine dioxide decontaminant (a) or sodium hypochlorite 0.5% decontaminant (b) in human skin keratinocyte cells. Just-confluent keratinocyte cultures were treated with varying decontaminant concentrations diluted in culture medium and incubated for 1, 4 or 24 hours before being removed and replaced with fresh culture medium containing no decontaminant. Cytotoxicity was determined at 24 hr (dotted bars) using the alamarBlue^TM^ cytotoxicity assay. The medium containing the dye was then removed and replaced with fresh culture medium, and cytotoxicity was determined again 24 hr later, for a total incubation time of 48 hours (solid bars). Data is expressed as the percentage of decontaminant needed to yield an LC_50_ with the keratinocyte culture after 1, 4, and 24hr exposure to the decontaminant. Results represent the mean +/- SD of four different experiments.

The concentration of sodium hypochlorite required to kill 50% of the human skin keratinocytes was not significantly different if the exposure time was one, four or 24 hours (Fig 4). In contrast, the toxicity of the chlorine dioxide product increased 4–6 fold as the exposure time increased from one to 24 hours (Fig 4). Although at exposures of 24 hours the concentration of the chlorine dioxide product was 20 fold higher compared to that of sodium hypochlorite to achieve an LC50. This result was consistent whether the toxicity to the skin cells was assessed at 24 or 48 hr (Fig 4).

## Discussion

Personal decontamination is defined as the reduction or removal of contaminants (chemical, biological or radiological; CBR) from oneself by either physical means or chemical neutralization [25, 30]. The development of a personal decontaminant is a complex task that requires achieving many criteria, including but not limited to: broad spectrum effectiveness, safety, application efficacy, rapid action, stability, cost, non-irritating and hypoallergenicity [25]. When addressing CBR agents, specific problems exist within each agent class with respect to ease of decontamination. Within the realm of biological decontamination, endospores such as *B*. *anthracis* are especially resistant to a number of commonly used decontaminants and decontaminating strategies [13, 24].

We used two strains of *B*. *anthracis* to assess the relative efficacy, and the toxicity of a decontaminant utilizing chlorine dioxide as the active ingredient, compared to sodium hypochlorite commonly used for biological decontamination. Experiments in which both strains of *B*. *anthracis* were suspension tested, showed that both sodium hypochlorite and the chlorine dioxide decontaminants were effective in reducing the concentration of spores in solution. In the non-virulent Sterne strain of *B*. *anthracis*, both decontaminants were similarly effective in reducing spore numbers after 10 minutes of exposure to below the limit of detection. However, at 5 minutes of contact time, the chlorine dioxide product was more efficacious (Fig 1). This was also noted with the virulent Ames strain; although this strain was more resistant to both decontaminants especially sodium hypochlorite at five minutes of contact time. This difference in susceptibility to decontamination between the two strains has been noted previously [31–33]. Other studies have shown that the differences between strains are minor and dependent on the type of disinfectant used [34, 35]. Results from our work may suggest that a small population of spores are resistant to disinfection, regardless of the starting concentration of spores or the strain of *B*. *anthracis* used, this difference is only for a short duration as both sporicides reduced spore loads to below detectable range after less than ten minutes of contact time. Experiments where the decontaminant was quenched using D/E broth showed that after 10 minutes Sterne spore loads were below the detectable limit confirm that both decontaminants are able to effectively kill spores after an appropriate contact time. After five minutes of contact time quenched samples had larger concentrations of viable spores compared to non-quenched samples.

Decontamination experiments in which isolated swine skin was used as a simulant for human skin compared the efficacy of the chlorine dioxide product against soapy water. Using a methodology predicated on guidelines for decontaminating individuals exposed to anthrax, with soapy water [13, 22] ~ 60 cm^2^ pieces of swine skin were heavily contaminated with the Sterne strain of *B*. *anthracis* and then “decontaminated” with the chlorine dioxide decontaminant or soapy water and gently scrubbed with a toothbrush. As expected, although soapy water reduced the spore count on the skin, considerable spore numbers were still detectable not only on the skin, but also on the scrub brushes and within the decontaminant washings. In contrast, decontamination of the swine skin with the chlorine dioxide decontaminant resulted in no viable spores being detected in the skin samples, the brushes or the washings. Water or soapy water is recommended as the decontaminant of choice in mass casualty situations [22, 23]. However, the major problem with this approach is that large quantities of contaminated water are produced, with the associated dangers of cross-contamination and, as in the case here of *B*. *anthracis*, poor removal of the agent from the skin.

Our final studies assessed the relative toxicities of sodium hypochlorite and the chlorine dioxide product; strong decontaminants are often associated with increased toxicity compared to milder products. Early passage cultures of human skin keratinocytes, the primary metabolic and protective components of the skin, were used to estimate the relative toxicities of the two decontaminants. Different exposure regimens were used, ranging from one to 24 hours of contact between the cells and the decontaminants, and using concentrations (v/v) of 0.02% to 10%. The toxicity of the sodium hypochlorite was much greater than that of the chlorine dioxide decontaminant in every case, being up to two orders of magnitude more toxic. In addition, the toxicity of the sodium hypochlorite was expressed maximally by one hour, indicating that even very short exposures below those tested here, may still produce injury. In contrast, the very low toxicity (~10%; v/v) of the chlorine dioxide product after one hour of contact increased only slowly over the next 23 hours, suggesting that reducing the exposure time should decrease the likelihood of skin injury. Studies such as this, where “naked” cells in culture are exposed to the test agent, should provide a conservative estimate of tissue injury, especially to intact skin, where the outer cornified epidermal layers are already dead and serve almost exclusively as a protective barrier.

This paper demonstrates that a chlorine dioxide based decontaminant is more effective and faster acting than 0.5% sodium hypochlorite at reducing *B*. *anthracis* spore numbers in solution. The main antimicrobial action of chlorine dioxide is the oxidation of bacterial cellular constituents including cell membranes by altering or disrupting the permeability of bacterial cell wall or viral envelope, and has been reported as 20 times more efficient than chlorine at killing *E*. *coli* [36].

Future work could include testing the decontaminant with a simulated organic load as this can have an impact on the efficacy of some disinfectants [37].Chlorine dioxide decontaminants have been shown to be effective in reducing numerous bacteria species including *Clostridium difficile*, evaluating this chlorine dioxide product as an effective tool in reducing *C*. *difficile* loads on skin would be an important area for further experimentation.

In summary, we report that a stabilized liquid formulation of chlorine dioxide is an effective sporicidal agent, and reduced *B*. *anthracis* spore counts by >5 logs in five minutes or less in suspension. Decontamination of isolated swine skin by this decontaminant was more effective than soapy water. The toxicity of this chlorine dioxide based decontaminant was less (two orders of magnitude) than sodium hypochlorite in human skin cell cultures. Decontaminant products utilizing this chemistry should exhibit high efficacy against sporulating organisms and low skin toxicity.

## Supporting Information

S1 File Decontamination Efficacy Data. (PDF)
