# Supplementary material for: Decontamination Efficacy and Skin Toxicity of Two Decontaminants against Bacillus anthracis
Source: PLoS One. 2015 Sep 22;10(9):e0138491. doi: 10.1371/journal.pone.0138491 (PMC4578770; doi:10.1371/journal.pone.0138491)
Supplement: S1 File — (PDF) [file pone.0138491.s001.pdf]

Microorganism	Test substance	Contact time	Replicate 1	Replicate2	Replicate 3
B. anthracis Stern Endospores	Product	Sb Ex2	1.54E+07	1.41E+07	1.60E+07
		Sb Ex1	1.16E+07	1.40E+07	8.80E+06
		Sb Ex3	1.28E+07	1.35E+07	1.57E+07
		T2 Ex2	7.00E+04	6.20E+04	3.60E+04
		T2 Ex1	2.80E+04	3.00E+04	3.00E+04
		T2 Ex3	1.45E+05	1.14E+05	9.80E+04
		T5 Ex2	TFTC (2)	TFTC (3)	TFTC
		T5 Ex1	TFTC	TFTC	TFTC (1)
		T5 Ex3	TFTC	TFTC	TFTC
		T10 Ex2	TFTC	TFTC	TFTC
		T10 Ex1	TFTC	TFTC	TFTC
		T10 Ex3	TFTC	TFTC	TFTC
		T30Ex3	TFTC	TFTC	TFTC
		T30Ex3	TFTC	TFTC	TFTC
		T30Ex3	TFTC	TFTC	TFTC
	PBS	0 minutes	1.47E+07	1.43E+07	1.77E+07
		0mins	1.09E+07	1.43E+07	1.16E+07
		0 mins	1.48E+07	1.40E+07	1.31E+07
		2 minutes	1.44E+07	1.51E+07	1.74E+07
		2mins ex1	1.40E+07	1.28E+07	9.60E+06
		2 mins ex3	1.61E+07	1.31E+07	1.50E+07
		5 minutes ex2	1.32E+07	2.02E+07	1.56E+07
		5mins ex1	9.10E+06	1.12E+07	1.11E+07
		5 mins ex3	1.21E+07	1.81E+07	2.04E+07
		10minutes ex2	1.63E+07	1.50E+07	1.53E+07
		10mins ex1	1.14E+07	1.18E+07	8.90E+06
		10 mins ex3	1.58E+07	1.53E+07	1.57E+07

Microorganism	Test substance	Contact time	Replicate 1	Replicate2	Replicate 3
B. anthracis Ames Endospores	Product	Percontact Sb Ex1	8.60E+06	8.70E+06	8.30E+06
		Percontact Sb Ex2	7.60E+06	6.80E+06	7.20E+06
		Percontact Sb Ex3	8.00E+06	8.30E+06	7.80E+06
		T2 Ex1	4.00E+03	8.00E+03	1.10E+04
		T2 Ex2	1.00E+04	8.60E+04	4.00E+04
		T2 Ex3	1.00E+03	8.00E+04	7.00E+04
		T5 Ex1	TFTC	TFTC	TFTC(8)
		T5 Ex2	TFTC	1.50E+01	TFTC
		T5 Ex3	TFTC	1.20E+01	TFTC
		T10 Ex1	TFTC	TFTC	TFTC
		T10 Ex2	TFTC	TFTC	TFTC
		T10 Ex3	TFTC	TFTC	TFTC
		T30 Ex1	TFTC	TFTC	TFTC
		T30 Ex2	TFTC	TFTC	TFTC
		T30 Ex3	TFTC	TFTC	TFTC
	PBS	30 minutes ex1	7.60E+06	8.60E+06	8.30E+06
		30mins ex2	8.00E+06	8.20E+06	8.40E+06
		30 mins ex3	9.20E+06	8.20E+06	8.50E+06

**Microorganism**	**Test substance**	**Contact time**	**Replicate 1**	**Replicate 2**	**Replicate 3**
B. anthracis Stern Endospores	Bleach	Percontact Sb Ex1	6.50E+06	6.60E+06	7.30E+06
		Percontact Sb Ex2	9.50E+06	1.09E+07	1.06E+07
		Percontact Sb Ex3	1.54E+07	1.41E+07	1.60E+07
		T2 Ex1	3.00E+04	1.80E+04	3.80E+04
		T2 Ex2	1.00E+04	1.00E+04	0.00E+00
		T2 Ex3	7.00E+04	6.20E+04	3.60E+04
		T5 Ex1	4.90E+01	1.79E+02	6.30E+01
		T5 Ex2	TFTC	TFTC	TFTC
		T5 Ex3	TFTC (7)	30	TFTC
		T10 Ex1	TFTC	TFTC	TFTC
		T10 Ex2	TFTC	TFTC	TFTC
		T10 Ex3	TFTC	TFTC	TFTC
		T30	TFTC	TFTC	TFTC
		T30	TFTC	TFTC	TFTC
		T30	TFTC	TFTC	TFTC
	PBS	0 minutes ex 2	1.09E+07	1.13E+07	1.01E+07
		0.5 minutes ex 1	9.20E+06	1.00E+07	1.11E+07
		0 minutes ex 3	1.47E+07	1.43E+07	1.77E+07
		0 minutes ex 4	1.48E+07	1.40E+07	1.31E+07
		2 minutes ex 2	1.15E+07	1.07E+07	1.03E+07
		2 minutes ex 3	1.74E+07	1.51E+07	1.74E+07
		2 minutes ex 4	1.61E+07	1.31E+07	1.50E+07
		5 minutes ex 2	6.30E+06	6.90E+06	7.20E+06
		5 minutes ex 3	1.32E+07	2.02E+07	1.56E+07
		5 minutes ex 4	1.21E+07	1.81E+07	2.04E+07
		10 minutes ex 1	6.30E+06	7.80E+06	7.00E+06
		10 minutes ex 3	1.63E+07	1.50E+07	1.53E+07
		10 minutes ex 4	1.58E+07	1.53E+07	1.57E+07

Microorganism	Test substance	Contact time	Replicate 1	Replicate2	Replicate 3
B. anthracis Ames Endospores	bleach	Percontact Sb Ex1	7.50E+06	8.30E+06	8.70E+06
		Percontact Sb Ex2	8.30E+06	8.60E+06	8.30E+06
		Percontact Sb Ex3	8.50E+06	5.00E+05	5.00E+06
		T2 Ex1	4.00E+03	6.00E+03	8.00E+03
		T2 Ex2	1.00E+04	1.00E+04	1.00E+04
		T2 Ex3	3.00E+04	4.10E+04	3.20E+04
		T5 Ex 1	TFTC	3.00E+01	8.00E+01
		T5 Ex2	4.90E+02	8.70E+02	6.30E+02
		T5 Ex3	2.20E+02	3.40E+02	4.50E+02
		T10 Ex1	TFTC	TFTC	TFTC
		T10 Ex2	TFTC	TFTC	TFTC
		T10 Ex3	TFTC	TFTC	TFTC
		T30 Ex1	TFTC	TFTC	TFTC
		T30 Ex2	TFTC	TFTC	TFTC
		T30 Ex3	TFTC	TFTC	TFTC
	PBS	0 minutes ex1	8.20E+06	7.90E+06	5.50E+06
		0 minutes ex2	5.80E+06	8.50E+06	7.20E+06
		0 mins ex3	7.60E+06	7.20E+06	7.40E+06
		2 minutes ex1	6.50E+06	5.80E+06	6.40E+06
		5 minutes ex1	5.60E+06	6.20E+06	5.20E+06
		5 minutes ex 2	8.60E+06	8.70E+06	8.50E+06
		10minutes ex1	7.10E+06	5.00E+06	6.60E+06
		10 minutes ex2	7.80E+06	6.30E+06	6.70E+06
		10 minutes ex3	8.20E+06	8.40E+06	8.90E+06

Microorganism	Test substance	Contact time	Replicate 1	Replicate2	Replicate 3
B. anthracis Sterne	Product	Percontact Sb Ex1	1.42E+07	1.20E+07	1.32E+07
Endospores	Plus D/E broth	Percontact Sb Ex2	1.18E+07	2.40E+07	1.80E+07
		Percontact Sb Ex3	1.22E+07	1.78E+07	1.56E+07
		T2 Ex1	5.50E+03	4.20E+03	4.20E+03
		T2 Ex2	1.30E+04	5.00E+03	6.80E+03
		T2 Ex3	6.90E+03	3.80E+03	5.60E+03
		T5 Ex1	4.10E+01	1.22E+02	3.20E+02
		T5 Ex2	6.60E+02	6.00E+02	7.20E+02
		T5 Ex3	6.70E+01	2.70E+02	1.30E+02
		T10 Ex1	TFTC	TFTC	TFTC
		T10 Ex 2	TFTC	TFTC	TFTC
		T10 Ex3	TFTC	TFTC	TFTC
		T30 Ex 1	TFTC	TFTC	TFTC
		T30 Ex 2	TFTC	TFTC	TFTC
		T30 Ex 3	TFTC	TFTC	TFTC
	PBS	0 minutes ex1	6.00E+06	3.00E+06	7.40E+06
		10minutes ex2	3.60E+06	8.00E+05	5.40E+06
		30minutes ex3	2.12E+06	3.00E+06	4.20E+06
	Product + D/E broth	Product + D/E (30min)	3.00E+06	4.00E+06	6.00E+06
		Control	5.60E+06	5.47E+06	5.50E+06

Microorganism	Test substance	Contact time	Replicate 1	Replicate2	Replicate 3
B. anthracis Stern Endospores	bleach plus D/E Quench	Percontact Sb Ex1	1.42E+07	1.20E+07	1.30E+07
		Percontact Sb EX2	1.18E+07	2.40E+07	1.90E+07
		Percontact Sb Ex3	2.40E+07	2.80E+07	2.20E+07
		T2 Ex1	2.80E+04	3.70E+04	5.60E+04
		T2 Ex2	1.10E+04	5.50E+04	8.10E+04
		T2 Ex3	3.30E+04	2.70E+04	4.40E+03
		T5 Ex1	4.70E+02	7.00E+02	4.20E+02
		T5 Ex 2	3.20E+02	2.80E+02	8.90E+02
		T5 Ex3	4.50E+02	4.80E+02	5.30E+02
		T10 Ex1	TFTC	TFTC	TFTC
		T10 Ex2	TFTC	TFTC	TFTC
		T10 Ex3	TFTC	TFTC	TFTC
		T30 Ex 1	TFTC	TFTC	TFTC
		T30 Ex 2	TFTC	TFTC	TFTC
		T30 Ex 3	TFTC	TFTC	TFTC
	PBS	0 minutes ex1	6.00E+06	3.70E+06	7.40E+06
		10minutes ex2	3.60E+06	8.20E+05	7.20E+06
		30minutes ex3	2.12E+06	3.10E+06	6.10E+06
	Bleach + D/E	Bleach + D/E (30 min)	5.30E+06	4.90E+06	4.70E+06
		Control	5.20E+06	5.80E+06	5.30E+06
